# Analysis of *SDHD* promoter mutations in various types of melanoma

**DOI:** 10.18632/oncotarget.4665

**Published:** 2015-07-27

**Authors:** Simone L. Scholz, Susanne Horn, Rajmohan Murali, Inga Möller, Antje Sucker, Wiebke Sondermann, Mathias Stiller, Bastian Schilling, Elisabeth Livingstone, Lisa Zimmer, Henning Reis, Claudia H. Metz, Michael Zeschnigk, Annette Paschen, Klaus-Peter Steuhl, Dirk Schadendorf, Henrike Westekemper, Klaus G. Griewank

**Affiliations:** ^1^ Department of Ophthalmology, University Hospital Essen, University Duisburg-Essen, West German Cancer Center and the German Cancer Consortium (DKTK), Essen Germany; ^2^ Department of Dermatology, University Hospital Essen, University Duisburg-Essen, West German Cancer Center and the German Cancer Consortium (DKTK), Essen Germany; ^3^ Institute of Pathology, University Hospital Essen, University Duisburg-Essen, West German Cancer Center and the German Cancer Consortium (DKTK), Essen Germany; ^4^ Department of Human Genetics, University Hospital Essen, University Duisburg-Essen, West German Cancer Center and the German Cancer Consortium (DKTK), Essen Germany; ^5^ University Duisburg-Essen and the German Cancer Consortium (DKTK), Essen Germany; ^6^ Department of Pathology, Memorial Sloan Kettering Cancer Center, New York NY, USA; ^7^ Marie-Josée and Henry R. Kravis Center for Molecular Oncology, Memorial Sloan Kettering Cancer Center, New York NY, USA

**Keywords:** melanoma, SDHD, promoter mutations

## Abstract

**Objectives:**

Recently, recurrent mutations in regulatory DNA regions, such as promoter mutations in the *TERT* gene were identified in melanoma. Subsequently, Weinhold et al. reported *SDHD* promoter mutations occurring in 10% of melanomas and being associated with a lower overall survival rate. Our study analyzes the mutation rate and clinico-pathologic associations of *SDHD* promoter mutations in a large cohort of different melanoma subtypes.

**Methods:**

451 melanoma samples (incl. 223 non-acral cutaneous, 38 acral, 33 mucosal, 43 occult, 43 conjunctival and 51 uveal melanoma) were analyzed for the presence of *SDHD* promoter mutations by Sanger-sequencing. Statistical analysis was performed to screen for potential correlations of *SDHD* promoter mutation status with various clinico-pathologic criteria.

**Results:**

The *SDHD* promoter was successfully sequenced in 451 tumor samples. ETS binding site changing *SDHD* promoter mutations were identified in 16 (4%) samples, of which 5 mutations had not been described previously. Additionally, 5 point mutations not located in ETS binding elements were identified. Mutations in UV-exposed tumors were frequently C>T. One germline C>A *SDHD* promoter mutation was identified. No statistically significant associations between *SDHD* promoter mutation status and various clinico-pathologic variables or overall patient survival were observed.

**Conclusions:**

Melanomas harbor recurrent *SDHD* promoter mutations, which occur primarily as C>T alterations in UV-exposed melanomas. In contrast to the initial report and promoter mutations in the *TERT* gene, our analysis suggests that *SDHD* promoter mutations are a relatively rare event in melanoma (4% of tumors) of unclear clinical and prognostic relevance.

## INTRODUCTION

Melanoma continues to be a major health burden worldwide [1, 2]. Effective removal of the tumor at an early stage remains the only reliable curative treatment. Although an impressive number of new systemic therapeutic approaches have become available in recent years [3–9], the long-term outlook for patients with metastatic disease remains poor.

A number of landmark genetic studies, primarily focusing on analyzing protein coding genes, have identified a large number of recurrently mutated genes in melanoma [10, 11]. In contrast to previously recognized mutations such as *BRAF* and *NRAS*, most of these genes are mutated less frequently (i.e. *NF1*, *RAC1*, *ARID1A*, etc.); their function and the clinical implications of these mutations are still poorly understood.

Recent efforts have moved beyond focusing primarily on protein coding genes and have identified mutations in non-protein coding areas. Potentially the most relevant such mutation identified to date was the finding of *TERT* promoter mutations in a large proportion of melanoma samples (30–70%) [12, 13]. These mutations were found to generate novel transcription factor binding sites increasing telomerase expression and have been associated with poor prognosis [14]. In search of novel recurrent mutations that alter transcription factor binding sites, Weinhold et al. recently reported recurrent mutations of the succinate dehydrogenase complex subunit D (*SDHD*) promoter in melanoma [15].

*SDHD* is one of two mitochondrial transmembrane subunits of the four-subunit succinate dehydrogenase (SDH) protein. SDH is an enzyme with two important functions. First, it acts as part of the citric acid cycle converting succinate to fumarate. Succinate functions as an oxygen sensor in the cell and stimulates cell growth in a hypoxic environment, in particular by stabilizing hypoxia-inducible factor (HIF), which controls several genes involved in cell division and the formation of new blood vessels [16–18]. Loss of SDH enzyme activity can lead to abnormal hypoxia signaling, leading to proliferation and tumor formation. The second known function of SDH is oxidative phosphorylation, an important process for the cell's energy budget.

Mutations in *SDHD* have been described in gastrointestinal stromal tumor (GIST) [19], paraganglioma [20, 21] and pheochromocytoma [22, 23]. Promoter mutations of *SDHD* in 13 of 128 (10%) melanomas were recently described by Weinhold et al. in a genome-wide analysis screening for mutations in noncoding regulatory regions of the DNA [15]. All three described recurrent hotspot mutations in the *SDHD* promoter region substitute a cytosine for a thymine nucleotide (C>T). The mutations are located at chr.11:111,957,523 (TTCC>TTTC), chr.11:111,957,541 (TTCC>TTTC) and chr.11:111,957,544 (CTTCC>TTTCC). The TTCC response element is highly conserved for E26 transformation-specific (ETS) transcription factors. The mutations alter existing ETS binding motifs predicted to lead to a reduced expression of the *SDHD* gene.

The aim of our study was to further evaluate the incidence of *SDHD* promoter mutations in a large cohort of melanoma samples of various subtypes and to investigate associations of *SDHD* mutation status with clinico-pathologic variables and other common oncogenic mutations in melanoma, such as *BRAF*, *NRAS*, *KIT* and *TERT* promoter mutations.

## RESULTS

### Tumors and patients

The *SDHD* promoter was successfully sequenced in 451 melanoma samples available for analysis, including 223 non-acral cutaneous, 38 acral, 33 mucosal, 43 occult, 43 conjunctival and 51 uveal melanoma samples. Based on lack of detected *SDHD* promoter mutations (addressed below), the 51 primary uveal melanoma samples were excluded from further statistical analyses. The 400 non-uveal melanoma samples included 167 primary tumors, 158 metastases, 5 recurrences, 43 occult and 27 not-classified tumor samples. The samples originated from 230 male and 170 female patients with a median age of 60 years (range 12–90 years) and a median follow-up time of 30 months (range 0.3–375 months) The clinico-pathologic information is summarized in Table 1.

**Table 1:** Characteristics of all tumor samples in regard to *SDHD* promoter status (*n* = 400)

All samples								
	*Total (All samples)*			*SDHD* WT		*SDHD* mut		
		*N*	%	*N*	*%*	*N*	*%*	*P*
Sex	Female	170	42.5	163	*41*	7	*2.3*	0.98
	Male	230	57.5	221	*55*	9	*1.7*	
Age at Diagnosis	Median	60						
	Range	12–90						
	<=60 years	191	47.5	174	*44*	6	*1.5*	0.45
	>60 years	172	42.8	174	*44*	7	*1.8*	
	Missing data	39	9.7	36	*9*	3	*0.8*	
Mutant oncogene**	WT	150	40	145	*39*	5	*1.3*	0.89
	BRAF*	142	38	n = 134	*35*	8	*2*	
	NRAS*	82	22	79	*21*	3	*0.8*	
	*KIT*	4	1	4	*1*	0	*0*	
	TERT prom. WT (underscribt)	115	55	112	*53*	3	*1.4*	0.38
	*TERT* prom. mut	95	45	89	*42*	6	*2.9*	
*BRAF* and *NRAS*	Either mutant*	222	59	n = 211	*56*	11	*2.9*	0.42
	Both WT	154	41	149	*40*	5	*1.3*	
Stage at diagnosis^#^	I	46	12	44	*11*	2	*0.5*	0.88
	II	122	31	118	*30*	4	*1*	
	III	122	31	118	*30*	4	*1*	
	IV	29	7	27	*7*	2	*0.5*	
	Missing data	81	20	77	*19*	4	*1*	
Anatomic distribution of primary	Non-acral skin	223	56	214	*54*	9	*2.3*	0.9
	Acral	38	10	38	*9*	0	*0*	
	Mucosal	33	8	32	*8*	1	*0.3*	
	Occult	43	11	40	*10*	3	*0.8*	
	Eye (conj.)	43	11	42	*11*	1	*0.3*	
	Missing data	20	5	18	*5*	2	*0.5*	
Anatomic site of skin and acral tumors	Head & neck	n = 40	10%	n = 39	9.8%	1	*0.3*	0.5
	Upper limbs	23	6	22	*6*	1	*0.3*	
	Trunk	n = 94	24	n = 88	*22%*	n = 6	*1.5%*	
	Lower limbs	64	16	63	*16*	1	*0.3*	
	Acral	38	10	38	*10*	0	*0*	
	Occult	43	11	n = 40	*9*	3	*0.8*	
	Missing data	n = 22	6	n = 20	*5%*	2	*0.5*	
Histologic type	ALM	30	8	29	*7*	1	*0.3*	0.9
	LMM	4	1	4	*1*	0	*0*	
	NM	77	23	75	*19*	2	*0.5*	
	SSM	46	12	43	*11*	3	*0.8*	
	Unclassified	243	61	233	*58*	10	*2.1*	
Breslow thickness	Median	3						
	Range	0.1–55.0						
	0.01–1.00mm	36	9	34	*8,5*	2	*0.5*	0.58
	1.01–2.00mm	42	11	39	*10*	3	*0.8*	
	2.01–4.00mm	82	21	80	*20*	2	*0.5*	
	>4.00mm	93	24	91	*23*	2	*0.5*	
	Missing data	147	37	140	*35*	7	*1.8*	
Clark level (skin tumors only)	I	0	0	0	*0*	0	*0*	0.89
	II	5	2	5	*2*	0	*0*	
	III	37	17	34	*15*	3	*1.3*	
	IV	74	33	72	*32*	2	*0.9*	
	V	21	9	20	*9*	1	*0.5*	
	Unknown	86	22%	n = 83	*21%*	3	*1.3*	
Sample type sequenced	Primary	167	42	163	*41*	4	*1*	0.34
	Metastasis	158	40	149	*37*	9	*2.3*	
	Recurrence	5	1	5	*1*	0	*0*	
	Occult	43	11	40	*10*	3	*0.8*	
	Missing data	27	7	27	*7*	0	*0*	
Ulceration	Absent	62	16	59	*15*	3	*0.8*	0.48
	Present	88	22	87	*22*	1	*0.3*	
	Unknown	250	63	238	*60*	12	*3.1*	
SLN	Negative	78	20	75	*19*	3	*0.8*	0.97
	Positive	84	21	81	*20*	3	*0.8*	
	Not done	238	60	228	*57*	10	*2.5*	

WT = wild-type; mut = mutant; ALM = acral lentiginous melanoma; NM = nodular melanoma; SSM = superficial spreading melanoma; LMM = lentigo maligna melanoma; SLN = sentinel lymph node; conj. = conjunctival; prom. = promoter

*p*-values are derived from chi-squared or Fisher exact tests, as appropriate

# Staging according to the American Joint Committee on Cancer (AJCC) Melanoma Staging System 2009[37]

* 2 cases harbored a *BRAF* and a *NRAS* mutation.

** *BRAF, NRAS, KIT* were screened in *n* = 376; the *TERT* promoter in *n* = 210 cases

### Oncogene mutations

*BRAF* mutations occurred in 38% (142/376) of the tumor samples, including 128 (90%) V600E and 8 (6%) V600K. Additionally, individual (1%) V600G, V600D, K601N, K601E, G469A and D594N mutations were identified. *NRAS* mutations were found in 82 (22%) tumor samples, including 37 (45%) Q61R, 25 (25%) Q61K, 14 (17%) Q61L, 4 (5%) Q61H and 2 (1%) G12D mutations. *KIT* mutations were found in 4 (1%) cases. *TERT* promoter mutations occurred in 95 of 210 analyzed cases (45%), including 52 (55%) chr.5:1,295,250C>T, 31 (33%) chr.5:1,295,228C>T, 11 (12%) chr.5:1,295,242_1,295,243CC>TT and 1 (1%) chr.5:1,295,228_1,295,229CC>TT mutations (Table 1).

### *SDHD* promoter mutation analysis

**Figure 1:**
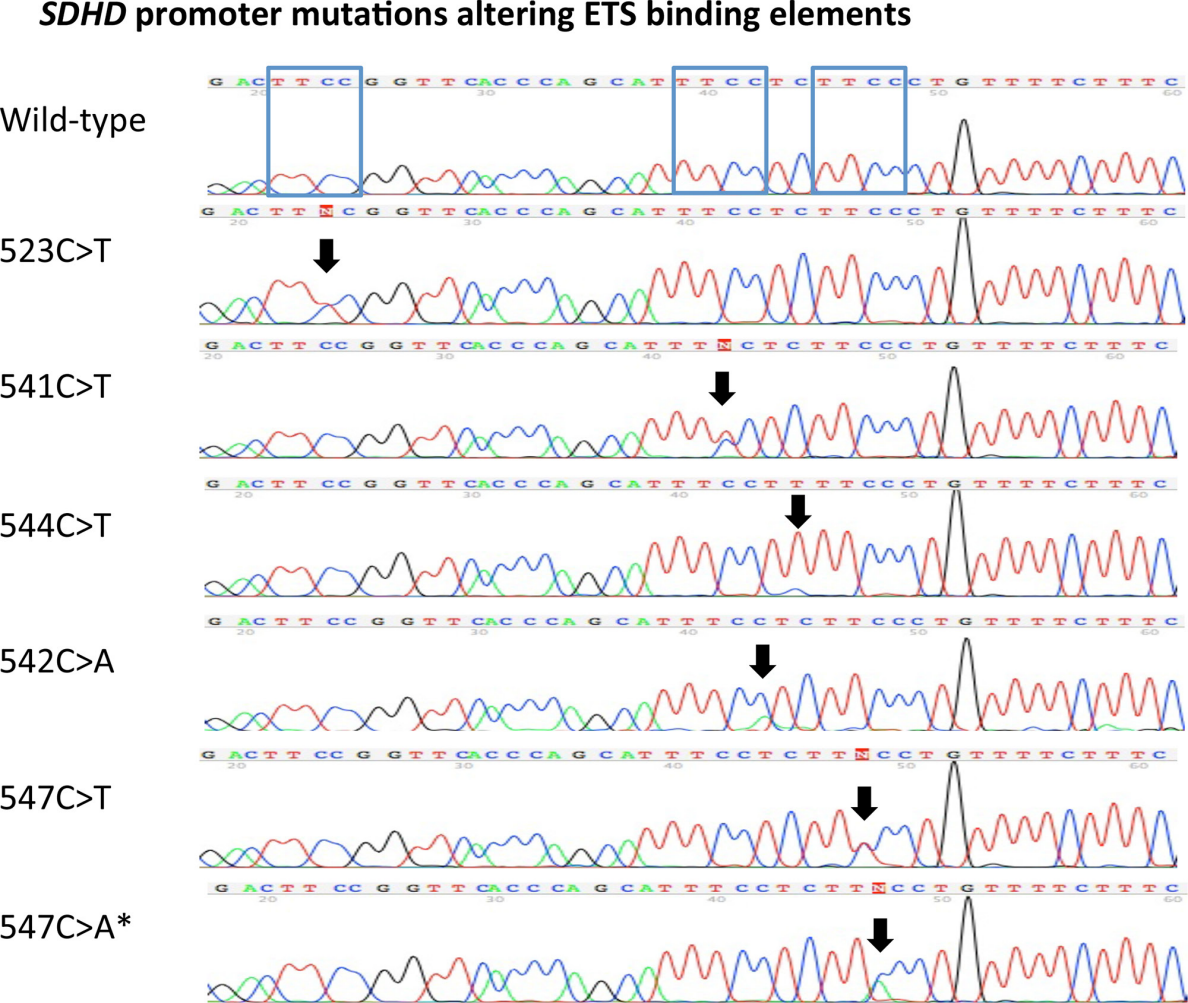
Recurrent *SDHD* promoter mutations altering ETS binding elements Sanger sequencing chromatograms of the identified recurrent *SDHD* promoter mutations located at chr.11:111,957,523C>T(TTCC>TTTC), chr.11:111,957,541C > T (TTCC>TTTC), chr.11:111,957,544C>T (CTTCC>TTTCC), chr.11:111,957,542C>A (TTCC>TTCA) and chr.11:111,957,547 (CTTCC>CTTTC and CTTCC>CTTAC) (according to human genome assembly [hg19]). The mutations locations are highlighted with black arrows. A wild-type promoter sequence for comparison is shown on the top. The blue boxes in the wild-type *SDHD* promoter sequence show the three different ETS binding elements. * signifies the mutation also identified in a germ-line sample.

ETS binding site affecting *SDHD* promoter mutations were identified in 16 of 400 tumors (4%), obtained from 7 female and 9 male patients. Eleven mutations were identified at the previously described locations [15]: chr.11:111,957,523 (TTCC>TTTC), chr.11:111,957,541 (TTCC>TTTC) and chr.11:111,957, 544 (CTTCC>TTTCC). Furthermore, we found five mutations occurring at two hotspots (Figure 1) located at chr.11:111,957,542 (TTCC>TTCA) and chr.11:111,957, 547 (CTTCC>CTTTC or CTTCC>CCTAC), which were not yet described, but also result in sequence alterations of the ETS-binding element (Figure 1). In the remaining manuscript, only the last three digits of the chromosome location nomenclature will be used for annotating the mutations location (i.e. 523C>T). The *SDHD* promoter mutations identified included 5 (1.3%) 523C>T cases, 3 (0.8%) 541C>T, 3 (0.8%) 542C>A, 3 (0.8%) 544C>T cases, 1 (0.3%) 547C>T and 1 (0.3%) 547C>A case (Table 2).

**Table 2:** *SDHD* promoter mutations in association to clinico-pathologic variables

Affecting ETS domains									Not affecting ETS domains				
SDHD promoter mutation		523C>T	541C>T	542C>A	544C>T	547C>T	547C>A	Total	529C>T	538C>A	550T>A	556C>T	Total
		*N*	*N*	*N*	*N*	*N*	*N*	*N*	*N*	*N*	*N*	*N*	*N*
Sex	Female	1	3	2	0	1	0	7	0	1	1	1	3
	Male	4	0	1	3	0	1	9	1	1	0	0	2
DNA type	Primary	3	1	0	0	0	0	4	0	0	0	0	0
	Metastasis	2	2	1	3	1	0	9	1	1	1	1	4
	Occult	0	0	2	0	0	1	3	0	0	0	0	0
	Missing data	0	0	0	0	0	0	0	0	1	0	0	1
Mutant oncogene	*BRAF*	2	1	2	2	1	0	8	0	0	1	0	1
	*NRAS*	1	0	0	1	0	1	3	0	0	0	0	0
	*KIT*	0	0	0	0	0	0	0	0	0	0	0	0
	*TERT* prom. mut	0	2	2	2	0	0	6	1	1	1	0	3
*BRAF* and *NRAS*	Either mutant	3	1	2	2	1	0	9	0	0	1	0	1
	Both WT	2	2	1	1	0	1	7	1	2	0	1	4
Clinical stage at diagnose^#^	I	1	0	0	1	0	0	2	0	0	0	0	0
	II	0	3	0	1	0	0	4	1	1	1	0	3
	III	2	0	1	1	0	0	4	0	0	0	1	1
	IV	0	0	1	0	0	1	2	0	0	0	0	0
	Missing data	2	0	1	0	1	0	4	0	1	0	0	1
Anatomic distribution of primary	Non-acral	3	3	1	2	0	0	9	1	1	1	1	4
	Acral	0	0	0	0	0	0	0	0	0	0	0	0
	Mucosal	0	0	1	0	0	0	1	0	0	0	0	0
	Occult	0	0	1	1	0	1	3	0	0	0	0	0
	Eye (conj.)	1	0	0	0	0	0	1	0	0	0	0	0
	Missing data	1	0	0	0	1	0	2	0	1	0	0	1
Germline	Analyzed samples	4	1	3	2	1	1	12/16	0	2	1	1	4/5
	Mutant (+/−)	−	−	−	−	−	+		−	−	−	−	

WT = wild-type; mut = mutant; prom. = promoter; conj. = conjunctival

# Staging according to the American Joint Committee on Cancer (AJCC) Melanoma Staging System 2009[37]

**Figure 2:**
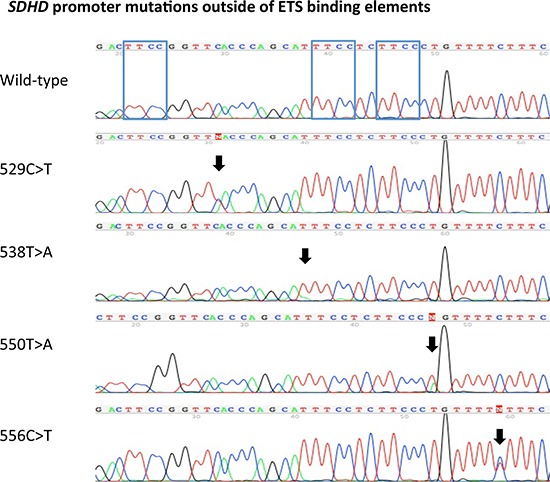
*SDHD* promoter mutations outside of ETS binding elements Sanger sequencing chromatograms of the identified *SDHD* promoter mutations located at chr.11:111,957,529 (TTCA>TTTA), chr.11:111,957,538 (TTTCC>ATTCC) chr.11:111,957,550 (CCCT>CCCA), and chr.11:111,957,556 (TTCT>TTTT) (according to human genome assembly [hg19]). The location of the mutations is highlighted with black arrows. The top chromatogram demonstrates a wild-type promoter sequence for comparison. The blue boxes in the wild-type *SDHD* promoter sequence show the three different ETS binding elements.

In addition to the ETS binding site affecting mutations, *SDHD* promoter mutations not affecting the ETS binding elements were identified in 5 tumor samples. These mutations included individual chr.11:111,957,529 (TTCA>TTTA), chr.11:111,957,550 (CCCT>CCCA) and chr.11:111,957,556 (TTCT>TTTT) mutations, as well as two chr.11:111,957,538 (TTTCC>ATTCC) mutations (Figure 2).

None of the 51 uveal tumor samples analyzed harbored a *SDHD* promoter mutation.

### Germline *SDHD* promoter analysis

**Figure 3:**
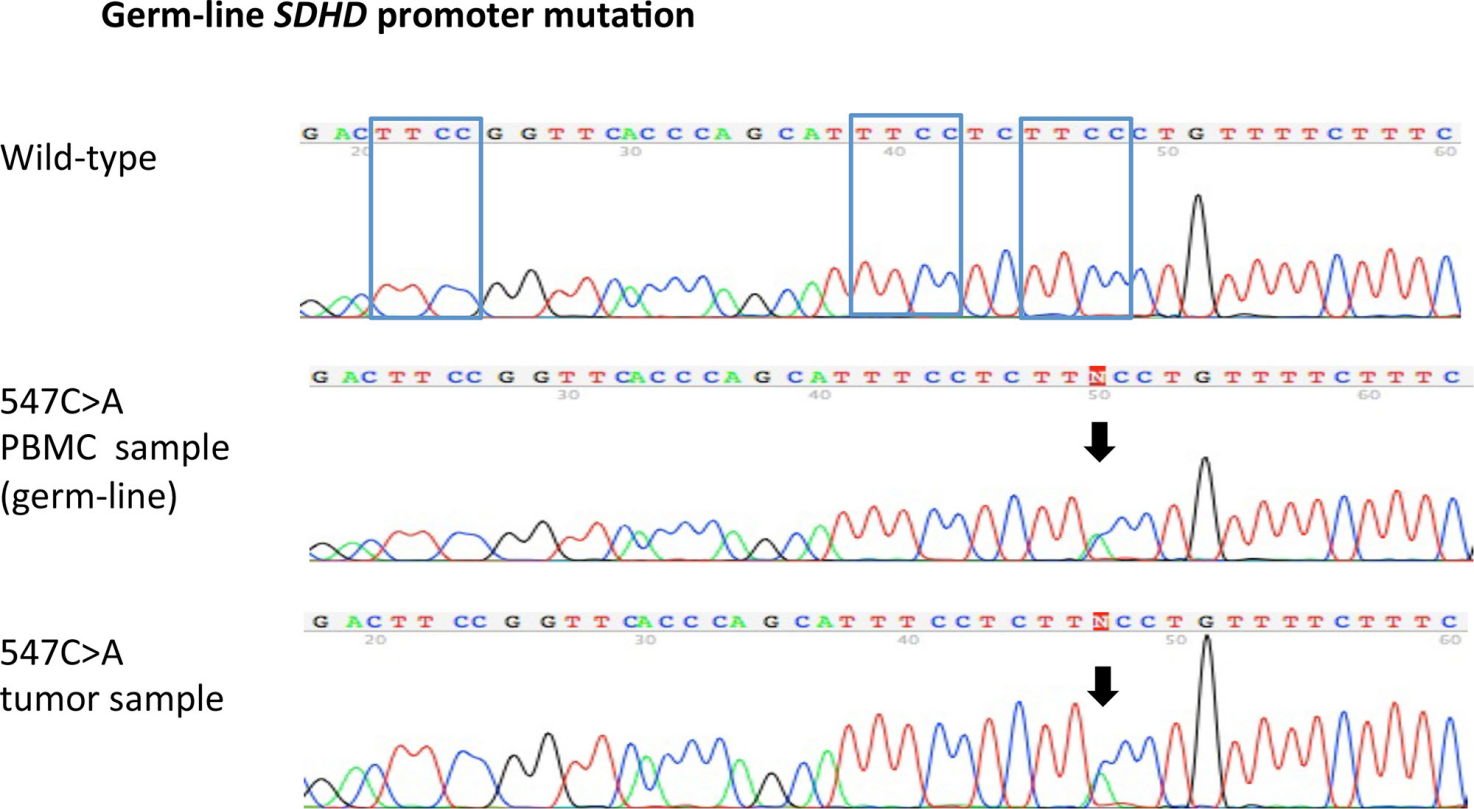
Germ-line *SDHD* promoter mutation Sanger sequence chromatograms show a chr.11:111,957,547C > A *SDHD* promoter mutation present both in the patient's melanoma sample as well as the germ-line. PBMC = peripheral blood mononuclear cells.

For tumors in which *SDHD* promoter mutations were identified, matching constitutional DNA from peripheral blood mononuclear cells was analyzed if available. This was the case in 12 of the 16 tumors with *SDHD* promoter mutations affecting ETS binding sites and 4 of 5 tumor samples with *SDHD* promoter mutations not affecting ETS binding sites. One of the ETS binding site affecting mutations (547C>A) was found to be present in the germline (Figure 3). In all other cases, *SDHD* promoter mutations detected in the tumor were not present in the constitutional DNA, confirming they were acquired somatically (Table 2).

### Clinico-pathologic correlations of *SDHD* promoter mutant tumors

For the analyses presented in the paper, only those *SDHD* promoter mutations affecting the ETS domains were deemed to be relevant (*n* = 16). However, no significant differences were observed when the samples harboring *SDHD* promoter mutations not affecting ETS binding domains were also included in the analysis (data not shown).

ETS binding site affecting *SDHD* promoter mutations were detected in 9 metastatic and 4 primary tumor samples (Table 1). Non-acral cutaneous melanomas harbored 9 of the 16 mutations (56%). One mutation each was found in a conjunctival and mucosal melanoma tumor sample. Three mutations occurred in occult tumor samples and in two cases anatomic site information was not available (Table 2). Of the 5 *SDHD* promoter mutations not affecting the ETS binding domains, 4 were in metastatic samples of non-acral cutaneous melanoma. No anatomic site information was available for the remaining case.

No statistically significant associations of *SDHD* promoter mutation status with available clinical parameters including stage at diagnosis, Breslow thickness, Clark level, presence of ulceration, histologic type and mutation status (*BRAF, NRAS, KIT, TERT* promoter) were identified (Table 1, Supplemental Table 1 + 2). This was the case regardless of whether selectively ETS binding site mutations (Table 1) or all identified mutations were considered (data not shown).

**Figure 4:**
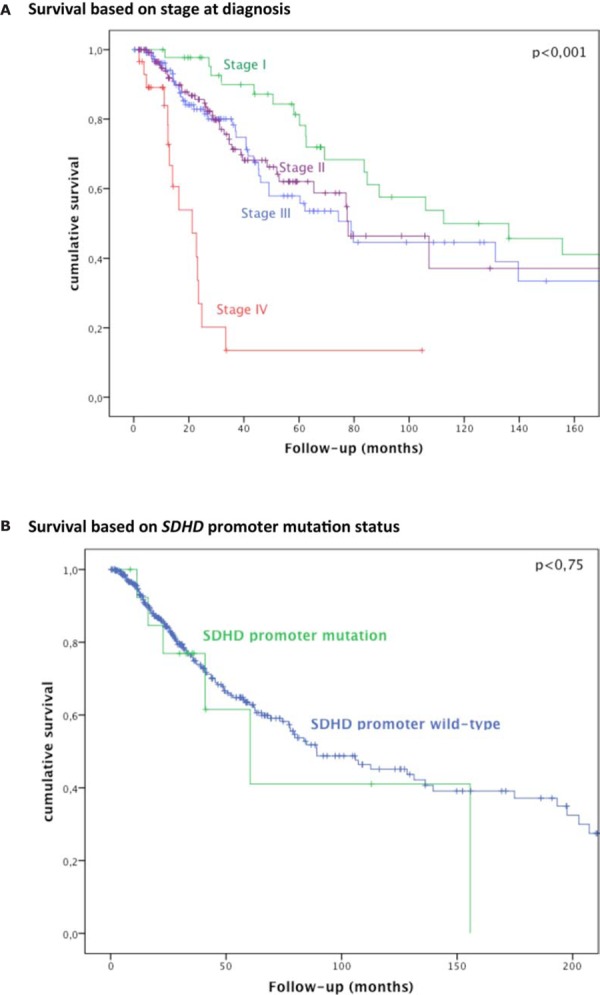
Survival based on stage at diagnosis and *SDHD* promoter mutation status Kaplan-Meier curves of overall survival in 400 patients with melanoma according to **A.** stage at diagnosis; **B.** *SDHD* promoter status (mutant vs wild-type).

In our cohort, 123 patients died, on average 52 months (range 2–375) after diagnosis. Six patients with *SDHD* promoter mutation died during the follow-up period. A statistically significant association between *SDHD* promoter mutation and overall survival was not found (Figure 4).

### *SDHD* promoter mutations in existing exome data

To explore the general frequency of *SDHD* promoter mutations and potential relevance for therapy resistance in melanoma, we re-analyzed exomes sequenced from 69 melanoma patients under MAPKi therapy [24]. DNA outside the targeted coding regions can be assessed if pulled down along with the targets during the capture process. Of 183 available exomes, sufficient coverage in the *SDHD* promoter region was obtained in 126 (69%) cases (min. 10x, max. 72.8x, average 22.7x). In the 92 tumor exomes with sufficient coverage we detected 4 *SDHD* promoter variants (4.3%, Supplemental Table 3). No mutations were detected in germline samples.

## DISCUSSION

In our study of a large cohort of ocular, cutaneous, mucosal and occult melanomas, *SDHD* promoter mutations affecting recurrent ETS binding elements were identified in only 4% (16/400) of the samples. Most mutations detected were at previously reported hotspots (*n* = 11) [15], with a C>T UV-signature [25, 26]. Additionally, 10 other mutations were identified, five of which altered the sequence of ETS transcription factor binding elements. Our study validates the finding of recurrent mutations in the *SDHD* promoter; however these mutations were considerably rarer than previously reported and showed no association with prognosis or the clinico-pathologic variables that were analyzed.

Most of the identified *SDHD* promoter mutations altering the TTCC element of the ETS transcription binding sites were found in one of the three previously described mutation hotspots located at chr.11:111,957,523, chr.11:111,957,541 and chr.11:111,957,544 (Figure 1). However, 5 additional mutations were identified at two previously undescribed hotspots, three mutations at Chr.11:111,957,542 (TTCC>TTCA) and two mutations at Chr.11:111,957,547 (CTTCC>CTTTC, CTTCC>CTTAC) (Figure 1). All of these newly identified mutations alter the ETS binding site core element (TTCC). In contrast, the previously reported recurrent 544C>T mutation is located just outside the core element (CTTCC>TTTCC, Figure 1). The altered nucleotide in this setting is conserved in a number of ETS transcription factors including ELF1 [15]. It would appear logical that the various mutations exert differing effects on *SDHD* gene transcription and protein translation. Detailed functional studies will be required to elucidate the extent to which these mutations differ in terms of their effect on transcriptional regulation of *SDHD*.

Mutation frequencies among melanoma subtypes did not vary greatly: non-acral cutaneous 4% (9 of 223), mucosal 3% (1 of 33), acral 0% (0 of 38), conjunctival 2% (1 of 43), occult 7% (3 of 43). The majority of ETS binding site altering *SDHD* promoter mutations (9 of 16 = 56%) were identified in the non-acral cutaneous melanoma. C>T mutations, which are a marker of UV-exposure, were observed only in these tumors. A single mucosal melanoma (1 of 33, 3%) harbored a 542C>A alteration; no mutations in acral (*n* = 38) melanomas were identified (Table 2). In contrast to C>T mutations, C>A alterations are not typical for UV induction. Additionally, no mutations were identified in uveal melanoma samples (*n* = 51), a tumor also lacking association with UV exposure [27]. The type of mutations identified does support *SDHD* promoter mutations in UV-exposed tumors (i.e. non-acral cutaneous) being primarily UV-induced, whereas the 542C>A mutation occurring in a mucosal, non UV-exposed melanoma probably developed in a UV-independent fashion. The overall lack of mutations in uveal melanoma further supports their being genetically distinct from cutaneous, mucosal and conjunctival melanoma [28–30].

The significance of the 5 *SDHD* promoter mutations identified outside of the three existing ETS binding elements (Figure 2) is unclear. Given that melanoma has a particularly high frequency of mutations [31], many of unclear functional relevance, it is possible that the identified alterations are simply passenger mutations. The only recurrent mutation, chr.11:111,957,538 (TTTCC>ATTCC), identified in two tumors, is located just outside of the ETS core element, similar to the previously described 544C>T mutation, however resulting in a different sequence (ATTCC versus TTTCC, respectively). Detailed functional studies will be required to determine the significance of these alterations. To exclude the possibility that these mutations might have (as yet unknown) functional importance, all statistical analyses were also performed including all 21 samples with *SDHD* promoter mutations (independent of their known effects on ETS binding sites). The results were similar in that no significant associations with various clinico-pathologic parameters including survival were observed.

The identification of a germline *SDHD* promoter mutation altering the ETS binding site is intriguing. The patient with the 547C>A germline mutation was a 79-old male presenting with stage IV disease upon initial diagnosis of an occult Melanom, which harbored an *NRAS* Q61K mutation (in addition to the 547C>A *SDHD* promoter mutation). The patient died of melanoma 4.5 months after diagnosis. Unfortunately, no detailed information on the patient and his family were available. Although the age of the affected patient argues against the role of this mutation in increasing the risk of melanoma, larger numbers of patients will be needed to adequately address this question.

There is a difference in mutation frequency observed between our study (∼4%) and the previous one by Weinhold et al. (10%). This could simply be due to differences in cohort characteristics and sample sizes (Weinhold et al. analyzed 128 samples). Another relevant factor may be the difference in experimental approaches applied. Weinhold et al. analyzed existing next generation sequencing data, searching for recurrent transcription factor mutations in the promoter region of genes, without sequencing validation. Our approach relied on targeted Sanger-sequencing. Both approaches have advantages and disadvantages. Sanger-sequencing does have a detection limit of ∼20%, and the potential to miss low-level mutations. On the other hand, next-generation sequencing approaches are not error-free, and can report incorrect results based on sequencing errors or various bioinformatic analytic hurdles.

To compare our Sanger sequencing results to NGS data, we explored an existing exome dataset detecting *SDHD* promoter variants in ∼4% of tumors. These variants were equally distributed in pre-treatment and recurrent tumors, showing no obvious association of *SDHD* variants with therapy resistance. Filter criteria for NGS analyses probably influenced the higher frequency of *SDHD* promoter variants in Weinhold's study. Their Bayesian methods identified the *SDHD* promoter as regionally recurrent in 5′UTRs (5 of 128 samples, with a FDR of 0.0016). However, the unique coverage at mutated positions and mutational frequency in the sequencing reads was not listed. Repeating our analysis without requiring a minimum of 2 unique reads to support a *SDHD* variant, we detected 11 variants in 92 tumors (12%). Strikingly, the additional variants were represented by a single sequencing read each and could likely represent false positives due to sequencing error. Even if one assumes some of the mutation calls could be correct, the question arises what biologic relevance such low frequency mutations may have. We believe that Sanger-sequencing remains a particularly robust form of sequence analysis and should be used whenever possible to confirm the presence of sequence variations before reporting newly-identified mutations.

No association of *SDHD* promoter mutation status with overall survival was seen (Figure 4). Admittedly, the number of mutated samples in our cohort is low (*n* = 16, or *n* = 21 if including *SDHD* promoter mutations not affecting ETS binding sites), meaning larger studies will be required to convincingly assess survival. However, Weinhold et al. reported a statistically significant (*p* = 0.005) survival difference with poorer prognosis for *SDHD* promoter mutations analyzing less mutant cases (*n* = 12). Given the discrepancy in our findings and taking into account Weinhold et al.′s relatively small sample size, we believe that the prognostic association of *SDHD* promoter mutations is yet to be unequivocally established and should be explored further in future studies.

It would be interesting to determine to which extent expression levels of SDHD protein are actually affected by *SDHD* promoter mutations. Considering the mutations are assumed to disrupt promoter binding sites, tumors with promoter mutations would be expected to show lower *SDHD* protein expression [15]. As the number of *SDHD* promoter mutated samples we found is very low, one can expect future studies will be required to screen large cohorts of tumors to allow a convincing statistical analysis of promoter mutation status and protein expression to be performed.

Overall, our study validates the finding of recurrent mutations in the *SDHD* promoter, which are enriched for mutations inactivating ETS transcription binding sites. However, in contrast to the initial report, the overall frequency of *SDHD* promoter mutation we identified is low (∼4%) and showed no association with poorer survival. Should these findings be validated in additional cohorts, they argue that compared to the much more frequent and prognostically relevant *TERT* promoter mutations, *SDHD* promoter mutations play a relatively minor role in melanoma.

## MATERIALS AND METHODS

### Sample selection

451 melanoma samples were obtained from patients treated in the Department of Dermatology or Ophthalmology of the University Hospital Essen, Germany. The samples included 223 non-acral cutaneous, 38 acral, 33 mucosal, 43 occult, 43 conjunctival and 51 uveal melanomas (Table 1). The study was performed in accordance with the guidelines put forth by the ethics committee of the University of Duisburg-Essen.

### Clinical and pathologic parameters

All clinical and pathologic parameters were obtained from patient records. The following parameters were assessed: sex, age, anatomic location of the tumor, pathologic stage, histologic subtype, Breslow thickness, Clark level, sentinel lymph node status, overall survival, and correlation with other gene mutations (incl. *BRAF*, *NRAS*, *KIT*, *TERT* promoter).

### DNA isolation and direct (Sanger) sequencing

Five ten-micrometer-thick sections were cut from paraffin-embedded tumor tissues and were deparaffinized. Genomic DNA was isolated using the QIAamp DNA Mini Kit (Qiagen, Hilden, Germany) according to the manufacturer's instructions. Polymerase chain reaction (PCR) was preformed to amplify the *SDHD* promoter region with the following primers: SDHD-F: ACC TTC CGA CAG CTG TGT TT, and SDHD-R: CTC AAG GTC ATC CAC CAA CC amplifying a 151-bp fragment. The PCR products were Sanger sequenced, as previously described [32]. For sequence-data analysis Chromas software was applied (version 2.01, University of Sussex, Brighton, UK). *BRAF* exon 15, *NRAS* exon 1 and 2, *KIT* exon 9, 11, 13, 17, 18 and the *TERT* promoter were PCR amplified and sequenced as previously described [32, 33]. Sequencing for *BRAF*, *NRAS* and *KIT* was generally performed sequentially; *NRAS* sequenced in *BRAF* wild-type samples, *KIT* in *BRAF* and *NRAS* wild-type samples. *SDHD* PCR and Sanger sequencing of peripheral blood mononuclear cells (PBMC) derived constitutional DNA was performed in 15 patients with *SDHD* promoter mutated tumor samples. DNA was isolated from PBMC, as described previously [34].

### Statistical analyses

Associations of *SDHD* promoter mutations and clinico-pathologic variables, such as age, sex, primary tumor location, TNM status, histologic type, mutation status (for *BRAF*, *NRAS*, *KIT* and *TERT* promoter mutations), Breslow thickness, Clark level, ulceration and sentinel lymph node status were explored using chi-square or Fisher exact tests as appropriate. A *p* value < 0.05 was considered statistically significant. For all statistical analysis, SPSS Statistics software (version 22.0; SPSS Chicago, IL) was applied.

### *SDHD* promoter analysis in existing exome data

We re-analyzed a dataset of 116 tumor and 67 germline exomes from 69 patients under MAPK inhibition (MAPKi) therapy [24] with respect to the *SDHD* promoter region. Bam files were indexed using samtools [35] and the hg19 human genome reference. Coverage was obtained using samtools-1.0 mpileup and the unix tools awk [36] and sed (http://www.gnu.org/software/sed/). Variant calls were performed using samtools-1.0 mpileup and bcftools query at positions 111,957,519 to 111,957,551 of chromosome 11 (32bp). A minimum average coverage of 10 unique reads across the 32bp region of the *SDHD* promoter was required and variants called when supported by at least 2 unique sequence reads.

## SUPPLEMENTARY TABLES



## Abbreviations

NGS: next generation sequencing
ETS: E26 transformation-specific
SDHD: Succinate dehydrogenase complex subunit D
