# Supplementary material for: Analysis of SDHD promoter mutations in various types of melanoma
Source: Oncotarget. 2015 Jul 27;6(28):25868–82. doi: 10.18632/oncotarget.4665 (PMC4694872; doi:10.18632/oncotarget.4665)
Supplement: Supplementary file 1 [file oncotarget-06-25868-s001.pdf]

SUPPLEMENTARY TABLES

Supplementary Table S1: Characteristics of primary and metastatic samples in regard to *SDHD* promoter status

		Primary Samples					Metastatic Samples				
		SDHD WT		*SDHD* mut		Total	*SDHD* WT		*SDHD* mut		Total
		N	*%*	*N*	*%*		*N*	*%*	*N*	*%*	
Sex	Female	70	*42*	2	*1,2*	72	66	*42*	4	*2,3*	70
	Male	93	*56*	2	*1,2*	95	83	*52*	5	*3,2*	88
Age at Diagnose	<=60 years	68	*40*	1	*0,6*	69	87	*55*	4	*2,3*	91
	>60 years	79	*48*	2	*1,2*	81	58	*37*	3	*1,9*	61
	Missing data	16	*10*	1	*0,6*	17	4	*2,5*	2	*1,2*	6
Mutant oncogene[#]	WT	72	*44*	3	*1,8*	75	49	*32*	2	*1,3*	51
	BRAF[*]	53	*33*	1	*0,6*	54	58	*38*	5	*3,3*	63
	NRAS[*]	31	*19*	0	*0*	31	36	*24*	2	*1,3*	38
	KIT	2	*1,2*	0	*0*	2	1	*0,6*	0	*0*	1
	WT	41	*60*	1	*1,4*	42	53	*51*	1	*1*	54
	TERT prom. mut	30	*42*	0	*0*	30	45	*43*	5	*4,8*	50
BRAF and *NRAS*[#]	Either mutant[*]	84	*52*	1	*0,6*	85	93	*61*	7	*5*	100
	Both WT	74	*46*	3	*1,9*	77	50	*33*	2	*1,3*	52
Stage at diagnosis	I	5	*3*	0	*0*	5	35	*22*	2	*1,3*	37
	II	59	*35*	1	*0,6*	60	51	*32*	3	*1,9*	54
	III	47	*28*	2	*1,2*	49	43	*27*	1	*0,6*	44
	IV	7	*4*	0	*0*	7	5	*3,2*	0	*0*	5
	Missing data	45	*27*	1	*0,6*	46	15	*10*	3	*1,9*	18
Anatomic distribution of primary	Non-acral	89	*54*	3	*1,8*	92	117	*74*	6	*3,8*	123
	Acral	18	*11*	0	*0*	18	17	*11*	0	*0*	17
	Mucosal	18	*11*	0	*0,6*	18	9	*6*	1	*0,6*	10
	Eye (conj.)	38	*23*	1	*0*	39	4	*2,5*	0	*0*	4

(*Continued*)

		Primary Samples					Metastatic Samples				
		***SDHD* WT**		***SDHD* mut**		**Total**	***SDHD* WT**		***SDHD* mut**		**Total**
		N	*%*	*N*	*%*		*N*	*%*	*N*	*%*	
	Missing data	0	*0*	0	*0*	0	2	*1,3*	2	*1,3*	4
Anatomic site of skin and acral tumors	Head & neck	26	*16*	0	*0*	26	24	*15*	1	*0,6*	25
	Upper limbs	12	*7*	0	*0*	12	10	*6*	1	*0,6*	11
	Trunk	43	*26*	2	*1,2*	45	43	*27*	5	*2,9*	48
	Lower limbs	17	*10*	1	*0,6*	18	46	*29*	0	*0*	45
	Genital	7	*4*	0	*0*	7	2	*1*	0	*0*	2
	Eye (conj.)	38	*22*	1	*0,6*	39	4	*3*	0	*0*	5
	Acral	18	*11*	0	*0*	18	17	*11*	0	*0*	17
	Missing data	2	*1,2*	0	*0*	2	3	*1,9*	2	*1,9*	2
Histologic type	ALM	10	*6*	0	*0*	10	16	*10*	1	*0,6*	17
	LMM	2	*1*	0	*0*	2	2	*1*	0	*0*	2
	NM	38	*23*	0	*0*	38	34	*22*	2	*1,2*	36
	SSM	16	*10*	2	*1,2*	18	27	*17*	1	*0,6*	28
	Unclassified	97	*58*	2	*1,2*	99	70	*54*	5	*3,6*	75
Breslow thickness	0.01-1.00mm	8	*5*	1	*0,6*	9	23	*13*	1	*0,6*	24
	1.01-2.00mm	18	*11*	0	*0*	18	19	*11*	3	*1,9*	22
	2.01-4.00mm	35	*21*	2	*1,2*	37	44	*26*	0	*0*	44
	>4.00mm	50	*30*	0	*0*	50	32	*19*	2	*1,3*	34
	Missing data	52	*31*	1	*0,6*	53	31	*20*	3	*1,9*	34
Clark level (skin tumors only)	II	1	*0,6*	0	*0*	1	3	*1,9*	0	*0*	3
	III	15	*9*	0	*0*	15	17	*11*	3	*1,7*	20
	IV	26	*15*	1	*0,6*	27	42	*27*	1	*0,6*	43
	V	8	*5*	0	*0*	8	12	*8*	1	*0,6*	13
	Unknown	113	*68*	3	*1,8*	116	75	*47*	4	*2,9*	79

(*Continued*)

		Primary Samples					Metastatic Samples				
		***SDHD* WT**		***SDHD* mut**		**Total**	***SDHD* WT**		***SDHD* mut**		**Total**
		N	***%***	***N***	***%***		***N***	***%***	***N***	***%***	
Ulceration	Absent	30	*18*	2	*1,2*	32	26	*17*	1	*0,6*	27
	Present	53	*32*	1	*0,6*	54	31	*20*	0	*0*	31
	Unknown	80	*48*	1	*0,6*	81	102	*61*	8	*5,2*	110
SLN	Negative	37	*22*	1	*0,6*	36	35	*22*	2	*1,3*	37
	Positive	46	*28*	2	*1,2*	48	32	*20*	1	*0,6*	33
	Not done	80	*48*	1	*0,6*	81	82	*52*	6	*4*	88

ALM = acral lentiginous melanoma; NM = nodular melanoma; SSM = superficial spreading melanoma; LMM = lentigo maligna melanoma; SLN = sentinel lymph node; WT = wild-type; prom = promoter; mut = mutant

[#]*BRAF, NRAS, KIT* were screened in *n* = 162 primary and *n* = 153 metastatic tumor samples; the *TERT* promoter was screened in *n* = 72 primary and *n* = 104 metastatic tumor samples

[*]1 metastatic case harbored a *BRAF* and a *NRAS* mutation

Supplementary Table S2: Associations between primary tumor location and clinico-pathologic variables of 400 melanoma samples.

		Acral		Non-acral Skin		Conjunctival		Mucosal		Occult		Missing data		Total
		N	*%*	*N*	*%*	*N*	*%*	*N*	*%*	*N*	*%*	*N*	*%*	
Sex	Female	19	5	85	21	20	6	21	5	17	4	8	*2*	170
	Male	16	4	136	34	23	6	12	3	26	7	17	*4*	230
Age	<=60 years	17	*4*	124	*31*	16	*4*	8	*2*	15	*4*	0	*0*	180
	>60 years	18	*5*	92	*23*	19	*5*	24	*6*	28	*7*	0	*0*	181
	Missing data	0	*0*	5	*0*	8	*2*	1	*0,2*	0	*0*	25	*6*	39
SDHD status	Wild-type	34	*9*	212	*53*	42	*11*	32	*8*	41	*10*	23	*6*	384
	Mutant	1	*0,3*	9	*2,3*	1	*0,3*	1	*0,3*	2	*0,5*	2	*0,5*	16
SDHD mutations	Wild-type	34	*9*	212	*53*	42	*11*	32	*8*	41	*10*	23	*6*	391
	523C>T	0	*0*	3	*0,8*	1	*0,3*	0	*0*	0	*0*	1	*0,3*	5
	541C>T	0	*0*	3	*0,8*	0	*0*	0	*0*	0	*0*	0	*0*	3
	544C>T	0	*0*	2	*0,5*	0	*0*	0	*0*	1	*0,3*	0	*0*	3
	542C>A	0	*0*	1	*0,3*	0	*0*	1	*0,3*	1	*0,3*	0	*0*	3
	547C>T	0	*0*	0	*0*	0	*0*	0	*0*	0	*0*	1	*0,3*	1
	547C>A[*]	0	*0*	0	*0*	0	*0*	0	*0*	1	*0,3*	0	*0*	1
Mutant oncogene[#]	*BRAF*	9	*2*	96	*26*	12	*3*	3	*0,8*	20	*5*	2	*0,5*	142
	KIT	0	*0*	0	*0*	1	*0,2*	3	*0,8*	0	*0*	0	*0*	4
	NRAS	10	*3*	51	*14*	7	*2*	1	*0,3*	11	*3*	2	*0,5*	82
	TERT prom.	4	*2*	62	*30*	13	*6*	4	*2*	12	*6*	0	*0*	95

(*Continued*)

		Acral		Non-acral Skin		Conjunctival		Mucosal		Occult		Missing data		Total
		N	*%*	*N*	*%*	*N*	*%*	*N*	*%*	*N*	*%*	*N*	*%*	
BRAF and *NRAS*	Either mutant	20	*5*	145	*39*	19	*5*	4	*1*	31	*8*	3	*1*	222
	Both WT	15	*4*	72	*19*	23	*6*	29	*8*	10	*3*	5	*1*	154
Clinical stage at diagnosis	I	3	*0,7*	40	*10*	0	*0*	3	*0,7*	0	*0*	0	*0*	46
	II	13	*3*	95	*24*	0	*0*	14	*3,5*	0	*0*	0	*0*	122
	III	16	*4*	73	*18*	0	*0*	7	*2*	26	*7*	0	*0*	122
	IV	2	*0,5*	6	*2*	0	*0*	5	*1*	16	*4*	0	*0*	29
	Missing data	1	*0,3*	7	*2*	43	*11*	4	*1*	1	*0,2*	25	*6*	81
Ulceration	Absent	7	*15*	50	*13*	0	*0*	4	*1*	1	*0,3*	0	*0*	62
	Present	15	*14*	65	*16*	1	*0,3*	7	*2*	0	*0*	0	*0*	88
	Missing data	13	*3*	106	*27*	42	*11*	22	*5*	42	*10*	25	*6*	250
Sentinel lymph node	Negative	10	*3*	60	*15*	0	*0*	7	*2*	1	*0,3*	0	*0*	78
	Positive	13	*3*	69	*17*	0	*0*	1	*0,3*	1	*0,3*	0	*0*	84
	Not done	12	*3*	92	*23*	43	*11*	25	*6*	41	*10*	25	*6*	238

*546C > A *SDHD* promoter mutation was found to present in the germline;

#*BRAF, NRAS, KIT* were screened in $n = 376$, the *TERT* promoter in $n = 210$ cases; 2 cases harbored a *BRAF* and a *NRAS* mutation

Supplementary Table S3: *SDHD* promoter variants called in whole exome data of 92 melanomas

Patient	Sample	Chr.	Position	Reference allele	Alternative allele	Average coverage	Read coverage	Reads alternative allele	Variant frequency	Core ETS site'TTCC' changed
Pat_22	post-treatment	11	111,957,539	T	A	25.53	31	2	0.065	Abrogated, TTTCC>TATCC
Pat_25	pre-treatment	11	111,957,547	C	T	21.16	27	8	0.296	Moved, TTCCC>TTTCC
Pat_25	post-treatment	11	111,957,547	C	T	22.47	30	11	0.367	Moved, TTCCC>TTTCC
Pat_52	pre-treatment	11	111,957,546	T	A	17.19	19	2	0.105	Abrogated, TTCC>TACC

The data was taken from [24]. A minimum coverage of 10 unique reads and at least 2 reads supporting a variant was required.
